# Regulating chemoresistance and cancer stemness: the CDH17-YAP pathway in distinct cellular states of lung cancer CTC clusters

**DOI:** 10.1186/s11658-025-00696-9

**Published:** 2025-02-24

**Authors:** Zujun Que, Dan Qi, Yun Yang, Wang Yao, Jiajun Liu, Yan Li, Yuanyuan Yu, Luyao Wang, Fangfei Li, Ge Zhang, Erxi Wu, Jianhui Tian

**Affiliations:** 1 Institute of Oncology, Shanghai Municipal Hospital of Traditional Chinese Medicine, Shanghai University of Traditional Chinese Medicine, Shanghai, 200071 China; 2 Clinical Oncology Center, Shanghai Municipal Hospital of Traditional Chinese Medicine, Shanghai University of Traditional Chinese Medicine, Shanghai, 200071 China; 3 Department of Neurosurgery and Neuroscience Institute, Baylor Scott & White Health, Temple, TX 76508 USA; 4 Department of Neurosurgery, Baylor College of Medicine, Temple, TX 76508 USA; 5 Law Sau Fai Institute for Advancing Translational Medicine in Bone and Joint Diseases, School of Chinese Medicine, Hong Kong Baptist University, Hong Kong, 999077 SAR China; 6 Institute of Precision Medicine and Innovative Drug Discovery, School of Chinese Medicine, Hong Kong Baptist University, Hong Kong, 999077 SAR China; 7 Institute of Integrated Bioinformedicine and Translational Science, School of Chinese Medicine, Hong Kong Baptist University, Hong Kong, 999077 SAR China; 8 College of Medicine, Texas A&M University, College Station, TX 77843 USA; 9 Irma Lerma Rangel College of Pharmacy, Texas A&M University, College Station, TX 77843 USA; 10 Department of Oncology, LIVESTRONG Cancer Institutes, Dell Medical School, The University of Texas at Austin, Austin, TX 78712 USA

**Keywords:** Lung cancer, Circulating tumor cells, Cancer stemness, Chemoresistance, CDH17-YAP pathway

## Abstract

**Background:**

Drug resistance in metastatic lung cancer significantly contributes to patient mortality. This study explores the role of circulating tumor cells (CTCs), the precursors to metastasis, in driving this resistance. We aim to delineate the unique biological traits of CTC clusters in lung cancer and elucidate the mechanisms underlying their resistance to chemotherapy.

**Methods:**

We used an ultralow adsorption plate to establish a CTC suspension culture system. Comparisons between adherent and suspension cultures of CTC-TJH-01 cells were made via Cell Counting Kit-8 (CCK-8), western blot, immunofluorescence, and flow cytometry assays to evaluate cell proliferation, drug resistance, and cancer stemness. The tumorigenicity, tumor growth rate, and drug resistance of the CTC clusters were assessed in nude mice. Transcriptomic and proteomic analyses were subsequently conducted to identify differentially expressed genes and proteins in CTC-TJH-01 cells cultured under adherent and suspension conditions. *CDH17* gene knockdown in CTC-TJH-01 cells was achieved through RNA interference, and hematoxylin and eosin (HE) staining, immunohistochemistry, and immunofluorescence assays were used to examine the pathological status of these cells.

**Results:**

CTC-TJH-01 cells in suspension formed cell clusters and exhibited decreased proliferation, tumorigenicity, and tumor growth, but increased cancer stemness and drug resistance. CDH17 protein expression was significantly upregulated in these clusters, activating the YAP/TAZ pathway. Knocking down CDH17 not only inactivated this pathway but also significantly increased cell proliferation activity and cisplatin sensitivity in CTC-TJH-01 clusters. Additionally, the tumor growth rate was correlated with cisplatin sensitivity. CDH17 knockdown notably promoted the growth of CTC-TJH-01 xenografts and enhanced their sensitivity to cisplatin, although no significant difference was observed compared with those in the control group.

**Conclusions:**

The results indicate that lung CTC clusters with stem cell-like properties exhibit chemoresistance, which is linked to an activated CDH17-YAP pathway. Additionally, the effectiveness of cisplatin is primarily observed in tumors with relatively high growth rates, highlighting the connection between tumor growth and sensitivity to chemotherapy.

**Graphical abstract:**

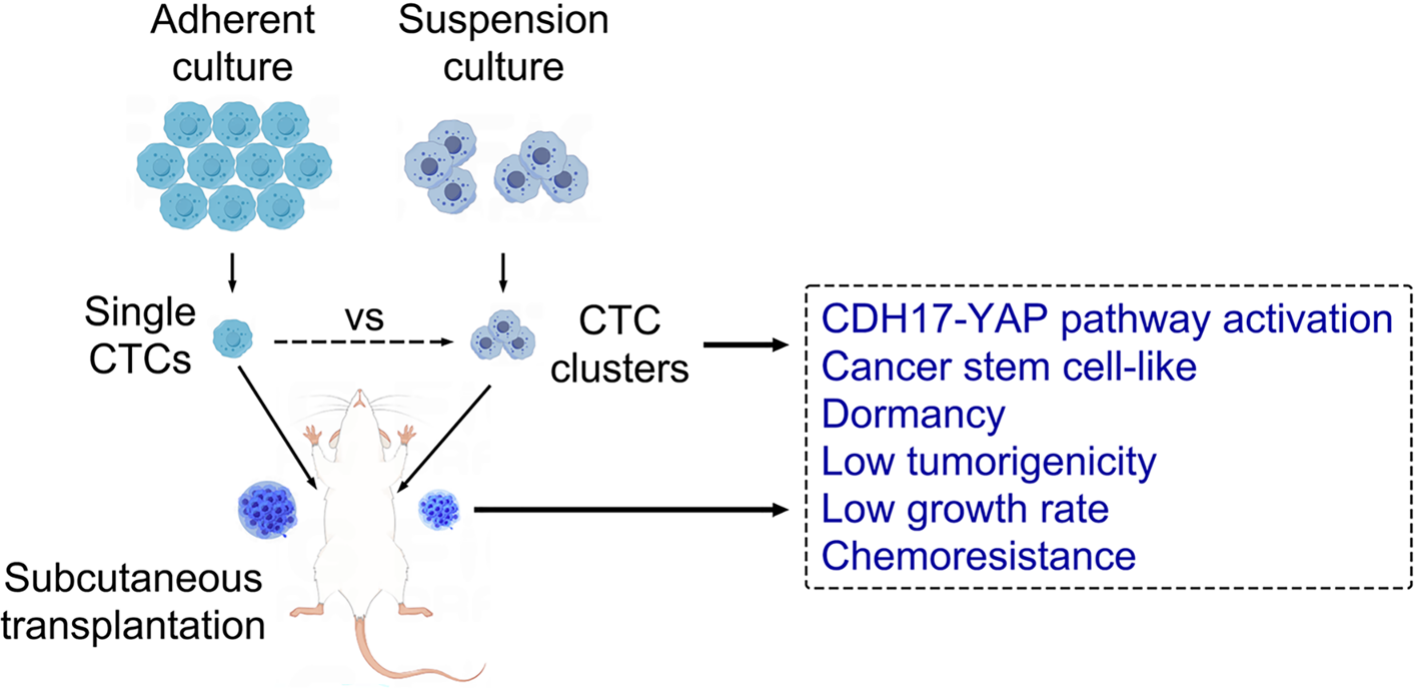

**Supplementary Information:**

The online version contains supplementary material available at 10.1186/s11658-025-00696-9.

## Introduction

Lung cancer, one of the most common malignant tumors, is a major global health threat [1]. The primary causes of death in lung cancer patients are tumor metastasis and drug resistance [2, 3]. Although molecular targeted therapy and immunotherapy have markedly improved disease-free survival (DFS) and overall survival (OS) in early postoperative patients with lung cancer [4, 5], their application is limited by strict eligibility criteria related to specific gene mutations and PD-L1 protein expression in tumor tissues. As a result, these therapies are not feasible for the majority of patients. Currently, chemotherapy is the mainstay of lung cancer treatment, yet it benefits only 5% of early postoperative patients [6]. Consequently, the 5-year overall survival rate for patients with lung cancer has remained at approximately 20%, with no substantial progress [7].

Circulating tumor cells (CTCs), which are found in peripheral blood, are pivotal in the distant metastasis of primary tumors [8]. Previous studies have demonstrated that CTCs can be detected in early-stage lung cancer [9]. Importantly, CTC clusters, which exhibit greater metastatic potential than solitary CTCs, are correlated with shorter DFS and OS [10]. These clusters, along with individual patient-derived CTCs, typically display cancer stemness, drug resistance, and dormancy [11]. Recent studies also indicate that CTCs and disseminated tumor cells (DTCs) in metastatic sites share the properties of cancer stem cells (CSCs) and dormant cells [12], which are crucial for their role in chemotherapy resistance [13]. Moreover, genome-wide single-cell copy number analysis by Seo et al. revealed a genetically clonal, yet phenotypically variable, CTC population in small cell lung cancer, emphasizing their strong cellular plasticity [14]. This plasticity, influenced by epigenetic regulation and interactions within the tumor microenvironment, is key to cancer progression [15]. CSC plasticity refers to the dynamic ability of tumor cells to switch between various cellular states, such as CSCs and non-CSCs, a process crucial for understanding treatment resistance, metastasis, and dormancy. This ability, often overlooked in bulk genomic analyses [16], allows cancer cells to adapt and transition through mechanisms, such as epithelial–mesenchymal transition (EMT) and mesenchymal–epithelial transition (MET), along with shifts between stem-like and non-stem-like states. Such phenotypic flexibility contributes to cancer proliferation, metastasis, and chemotherapy resistance, which is particularly evident in early postoperative patients with lung cancer. These findings highlight the critical need for more in-depth exploration of cancer stemness and drug resistance mechanisms during metastasis and the development of new therapeutic targets.

Cadherins, a family of adhesion molecules, are crucial for cell–cell adhesion and play significant roles in tumor metastasis. Specifically, cadherin-17 (CDH17), known as liver–intestine cadherin or human peptide transporter-1, is implicated in tumor invasion and metastasis [17], and its high expression in tumor tissues correlates with poor prognosis in patients with colorectal cancer [18]. In addition, the Hippo signaling pathway, which controls organ size by regulating cell proliferation and apoptosis, is often dysregulated in various cancers, contributing to tumorigenesis, metastasis, and drug resistance [19]. Studies have shown that the Hippo pathway effector YAP1 influences the morphological plasticity and metastasis of tumor cells [20, 21]. However, the role of CDH17 and the Hippo signaling pathway in the proliferation, metastasis, and drug resistance of CTC clusters in lung cancer has not been fully explored.

Building on our established research framework with CTC-TJH-01 cells [22, 23], we are dedicated to investigating the biological traits and molecular dynamics of CTCs in different cellular states of lung cancer CTC clusters. Our research focuses on determining the plasticity of tumor cells, namely the relationship between the proliferation rate of cells and their sensitivity to chemotherapy drugs. We aim to identify the key pathways regulating chemoresistance, cancer stemness, and tumor proliferation. This work aims to deepen our understanding of these critical mechanisms and pinpoint potential targets for antimetastatic therapeutic interventions.

## Methods and materials

### Reagents and antibodies

Cisplatin and paclitaxel were procured from MACKLIN (Shanghai, China). The CDH17 small interfering RNA (siRNA) and ribo*FECT*™ CP Transfection Kit were purchased from RIBOBIO (Guangzhou, China). The CDH17 overexpression plasmid and the control plasmid were purchased from GeneChem (Shanghai, China). The Lipofectamine™ 3000 transfection kit was purchased from Invitrogen (Carlsbad, CA, USA). Antibodies against ABCG2, ALDH1, CD44, SOX2, OTC-4, α-catenin, p-α-catenin, YAP/TAZ, p-YAP, and Ki-67 were obtained from Abcam (Cambridge, UK). Antibodies against GAPDH, CDH17, 14-3-3, caspase-3, cleaved-caspase-3, survivin, goat anti-mouse IgG-HRP, and donkey anti-rabbit IgG-HRP were obtained from Affinity Biosciences. Antibodies specific for CD133 were purchased from Miltenyi Biotec.

### Cell culture

Lung cancer patient-derived CTCs (CTC-TJH-01) were established by our laboratory as we previously reported [22, 23]. CTC-TJH-01 cells were cultured in F12K medium (Gibco, CA, USA). An ultralow adsorption cell culture plate (Corning, no. 3473) was used to establish the CTC-TJH-01 cell suspension culture system.

### Animals

We used 6-week-old male nude mice and NOD/SCID mice, which were obtained from GemPharmatech (Nanjing, Jiangsu), as the mouse models. The animals were housed under pathogen-free conditions in accordance with the Guide for the Care and Use of Laboratory Animals. All the animal experiments were approved by the Animal Ethical and Welfare Committee of Shanghai Municipal Hospital of Traditional Chinese Medicine, Shanghai University of Traditional Chinese Medicine (approval no. 2020-0014), in compliance with the guidelines of the Basel Declaration.

### Morphological observation

CTC-TJH-01 cells in suspension and adherent cultures were observed and photographed under an inverted microscope with a digital camera (Leica, Wetzlar, Germany).

### In vitro cell growth assays

CTC-TJH-01 cells (3000 cells/200 μl) were seeded in precoated or ultralow adsorption 96-well plates. Cell proliferation was assessed every 24 h using a Cell Counting Kit-8 (Dojindo, Shanghai, China).

### Cell cycle analysis

For the cell cycle analysis, CTC-TJH-01 cells were cultured in adherent media and suspensions for 48 h. Then the cells were digested, collected, stained with PI, and analyzed with a FACSVerse™ flow cytometer (BD Biosciences, CA, USA).

### Quiescent cancer cell analysis

CTC-TJH-01 cells were cultured in adherent media and suspensions for 48 h. Then the cells were digested and collected, and stained with PI and Ki-67 separately. Flow cytometry was subsequently used to analyze the proportion of Ki-67 negative cells in the G_0_/G_1_ phase, which were considered cancer cells in the quiescent stage.

### Drug sensitivity assays

CTC-TJH-01 cells (5000 cells/100 μl/well) were seeded in ordinary or ultralow adsorption 96-well plates. After overnight incubation, the cells were treated with cisplatin or paclitaxel for 24, 48, or 72 h, and cell viability was assessed via a CCK-8 assay.

### Apoptosis analysis

The annexin V-FITC/PI apoptosis assay was performed as previously described [24]. CTC-TJH-01 cells were seeded in ordinary or ultralow adsorption six-well plates. After overnight incubation, the cells were treated with cisplatin or paclitaxel for 48 h, and cell apoptosis was assessed via an annexin V-FITC/PI apoptosis assay (BD Biosciences, CA, USA).

### Western blot analysis

Western blotting was conducted as described previously [25]. Briefly, the cells were lysed, and the proteins were extracted. Then, 40 µg of protein were used for western blot analysis.

### Transfection

RNA interference assays were performed as described previously [26]. In brief, CTC-TJH-01 cells were seeded in ultralow adsorption 24-well plates. After 4 h, the cells were transfected with CDH17 siRNA, via a ribo*FECT*™ CP Transfection Kit (RiboBio, China). An unrelated, scrambled siRNA was used as a negative control. For the CDH17 plasmid, 2 μg of the CDH17 plasmid was transfected via a Lipofectamine™ 3000 transfection kit, as instructed by the manufacturer. An empty vector plasmid was used as a negative control.

### Immunofluorescence staining assays

In brief, CTC-TJH-01 cells were seeded in ultralow adsorption 6-well plates or laser confocal small dishes. After 48 h, the cells were first stained with CDH17, and then red fluorescent secondary antibody and DAPI. The expression of CDH17 was captured via a Leica TCS-SP8 laser confocal microscope.

### Transcriptomics, proteomics, and bioinformatics analysis

Briefly, CTC-TJH-01 cells were collected after adherent and suspension culture, after which the cells were sent to Sinotech Genomics (Shanghai, China) for RNA sequencing and Hangzhou Jingjie Biotechnology Co., Ltd (Zhejiang, China) for protein mass spectrometry. The overlapping differential genes and proteins were analyzed.

### Tumor growth assays

CTC-TJH-01 cells in suspension or adherent culture were injected subcutaneously into the right or left armpit of 6-week-old male nude mice with a density of 5 × 10^5^ cells (100 μl). The development and growth of the tumors were measured twice a week with a Vernier caliper. The tumor volumes were calculated using the formula: [sagittal dimension (mm) × cross dimension (mm)^2^]/2 and are expressed in mm^3^. The animals were euthanized once the volume of the tumor exceeded 2000 mm^3^. Approximately 9 weeks after inoculation, the mice were sacrificed, and the tumors were sectioned, stained with hematoxylin and eosin (HE) and subjected to immunohistochemistry.

### Immunohistochemistry assays

Subcutaneous tumors were fixed with 4% paraformaldehyde, embedded in paraffin, and sectioned. The tumor sections were stained with HE and antibodies against human CDH17 and Ki-67. The slides were scanned with an automatic digital pathological section scanner (KFBIO, Zhejiang, China).

### Statistical analysis

The significance of the differences was determined via Student’s *t*-test or one-way ANOVA. Kaplan–Meier analysis was employed for survival analysis and the differences in the survival probabilities were estimated using the log-rank test. All the statistical analyses were performed via GraphPad Prism 8.0 (GraphPad, San Diego, CA, USA). All experiments were performed at least in triplicate (*n* = 3). The data are expressed as the mean ± standard deviation (SD) or the mean ± standard error of the mean (SEM). The levels of statistical significance were set at *P < 0.05, **P < 0.01, and ***P < 0.001.

## Results

### CTC spheroid cells in suspension exhibit dormancy and CSC-like characteristics

**Fig. 1:**
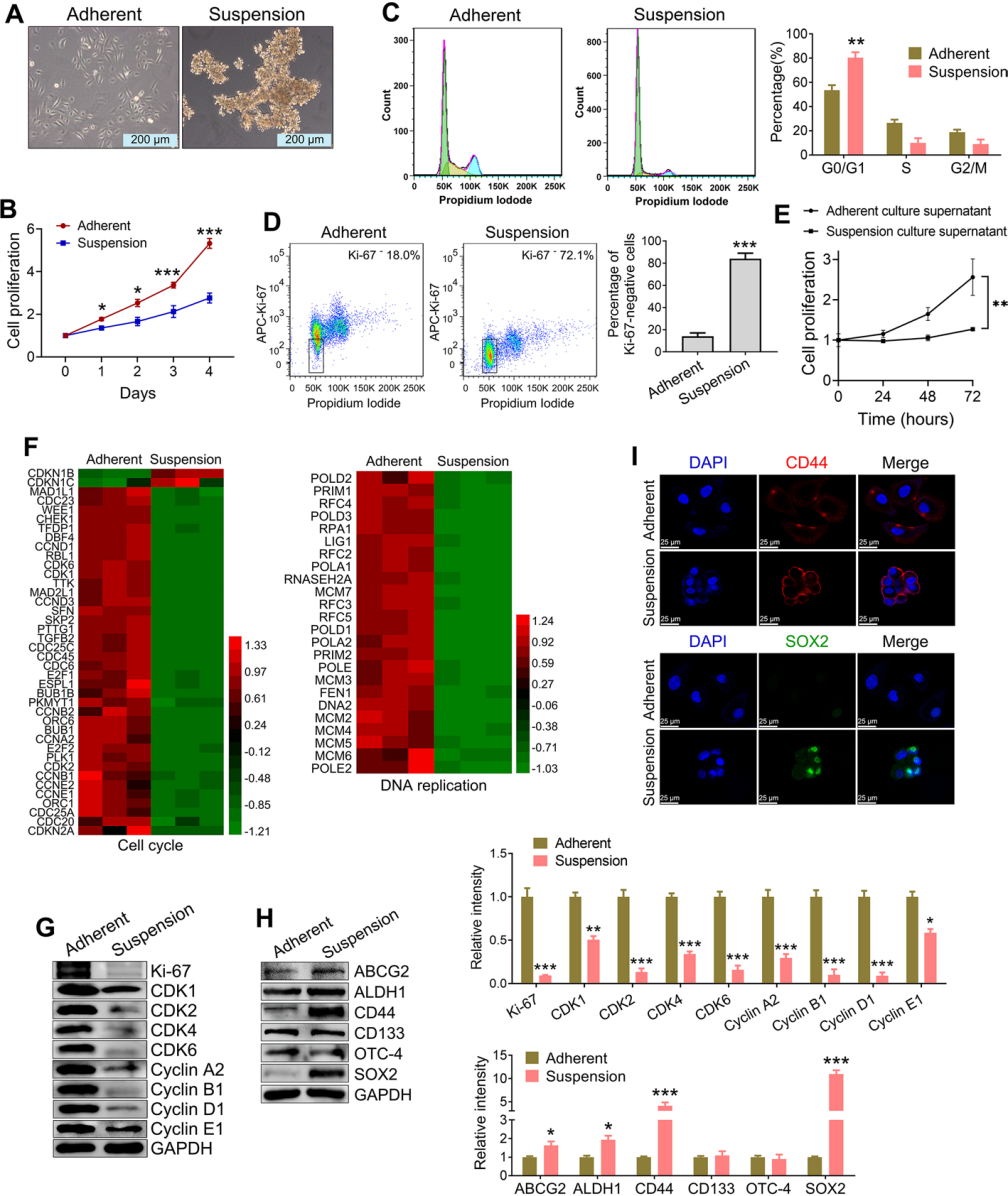
Comparison of the biological characteristics of CTC-TJH-01 cells in different growth states. **A** Morphological observation of the CTC-TJH-01 cells after adherence and suspension culture. Scale bar, 200 μm. **B** Comparison of the proliferation ability of adherent and suspension cultured CTC-TJH-01 cells. **C** Analysis of the cell cycle distribution of CTC-TJH-01 cells after adherent and suspension culture. **D** Flow cytometry analysis of the proportion of quiescent cells in adherent and suspension cultures of CTC-TJH-01 cells. **E** Comparison of the proliferative activity of adherent-cultured CTC-TJH-01 cells cultured with the supernatants of adherent-cultured and suspension-cultured CTC-TJH-01 cells. **F** Analysis of differential gene heatmaps related to the cell cycle and DNA replication signaling pathways in CTC-TJH-01 cells cultured in adherent and suspension cultures after transcriptome sequencing. **G** Expression of cell proliferation related proteins in adherent and suspension cultured CTC-TJH-01 cells. **H** Expression of cancer stem cell related proteins in adherent and suspension-cultured CTC-TJH-01 cells. **I** The expression of CD44 and SOX2 proteins in adherent and suspension-cultured CTC-TJH-01 cells by immunofluorescence staining. Scale bar, 25 μm. Each bar represents the mean ± SD of three separate experiments. *P < 0.05; **P < 0.01; ***P < 0.001

Our previous study successfully established the CTC line CTC-TJH-01 using CTCs from a patient with lung cancer [22, 23]. To further understand the unique cell biological characteristics of CTCs, we utilized our established patient-derived CTC line CTC-TJH-01 and established a suspension culture system using an ultralow adsorption cell culture plate. As shown in Fig. 1A, CTC-TJH-01 cells in suspension naturally aggregated to form clusters. Compared with that of adherent cultures, the proliferative activity of CTC-TJH-01 cells in suspension was significantly reduced (Fig. 1B), the cell cycle arrested in the G_0_/G_1_ phase (Fig. 1C), and the proportion of cancer cells in the quiescent stage increased significantly (Fig. 1D). Interestingly, we observed that the supernatant of the suspension-cultured CTC-TJH-01 cells inhibited the proliferative activity of the adherent-cultured CTC-TJH-01 cells (Fig. 1E). In addition, transcriptome sequencing data revealed significant downregulation of genes related to the cell cycle and DNA replication in suspension-cultured CTC-TJH-01 cells (Fig. 1F). We also confirmed that the expression of Ki-67 and cell cycle related proteins was significantly downregulated in suspension-cultured CTC-TJH-01 cells (Fig. 1G). Crucially, there was a notable increase in the expression of CSC markers such as SOX2, CD44, ABCG2, and ALDH1 in the suspension-cultured CTC-TJH-01 cells (Fig. 1H). Specifically, there was a marked increase in CD44 expression on the cell membrane and SOX2 expression in the nucleus (Fig. 1I). These findings collectively demonstrate that CTC clusters in suspension exhibit characteristics typical of CSCs.

### CSC-like CTCs have weaker tumorigenicity and tumor growth capacity in vivo

**Fig. 2:**
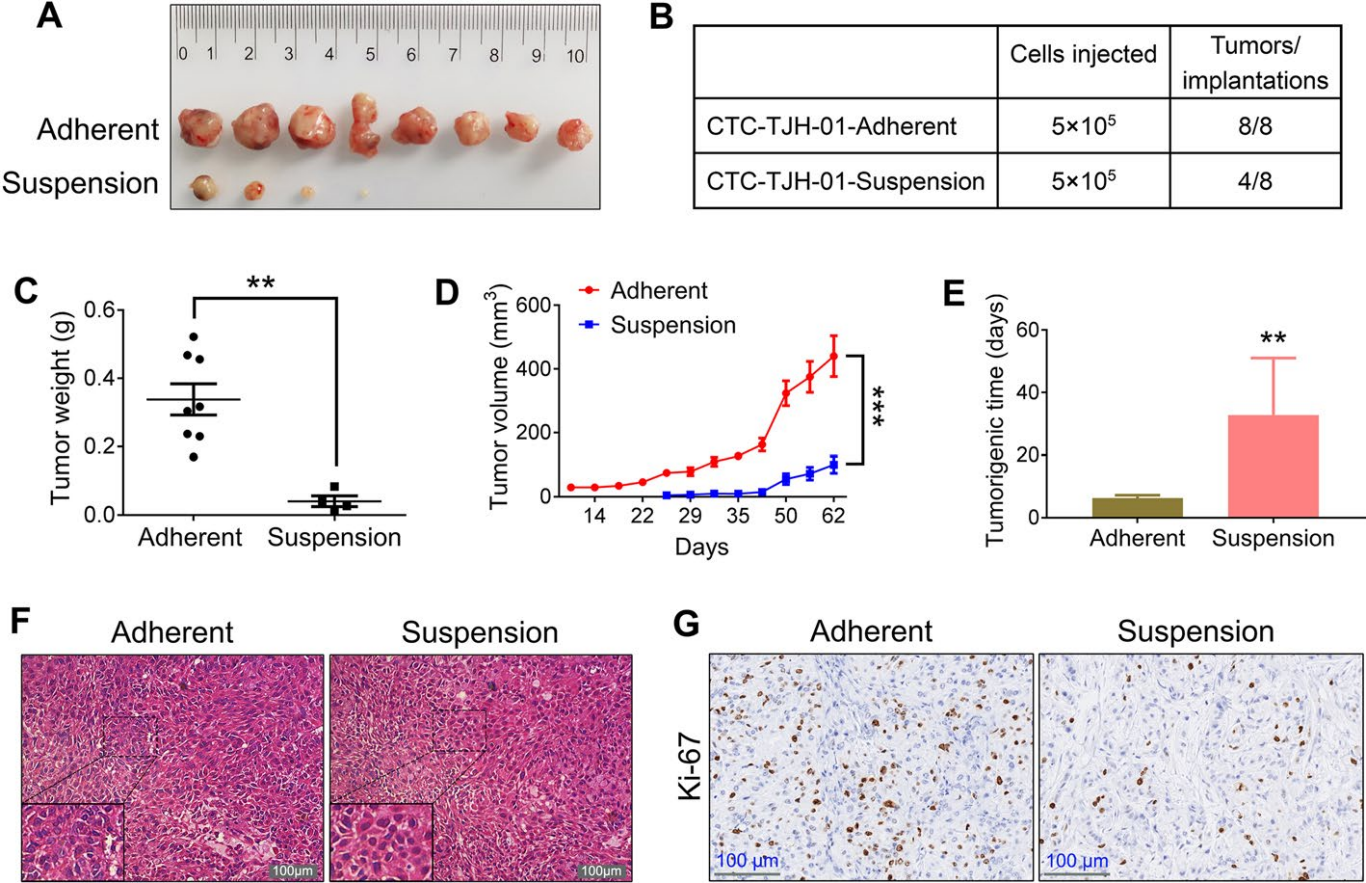
Comparison of the tumorigenicity of CTC-TJH-01 cells in adherent and suspension cultures in vivo. **A** A representative image of tumors excised 62 days after subcutaneous transplantation of adherent and suspension-cultured CTC-TJH-01 cells (5 × 10^5^ cells) into nude mice (*n* = 8). **B** Tumor-seeding ability of CTC-TJH-01 cells after suspension and adherent culture. **C** Tumor weight was measured with an electronic balance. **D** Growth curves of the tumors arising from the inoculation of adherent and suspension cultured CTC-TJH-01 cells. Tumor growth was measured with a digital caliper. **E** Tumorigenesis time of adherent and suspension cultured CTC-TJH-01 cells. **F** HE staining of adherent and suspension-cultured CTC-TJH-01 cell xenografts. Scale bar, 100 μm. **G** Immunohistochemical analyses of Ki-67 expression levels in adherent or suspension-cultured CTC-TJH-01 tumor tissues. Scale bar, 100 μm. Each bar represents the mean ± SEM of three separate experiments. *P < 0.05; **P < 0.01; ***P < 0.001

To assess the tumorigenicity and tumor growth capabilities of CSC-like CTCs in vivo, we subcutaneously transplanted equal numbers of adherent and suspension-cultured CTC-TJH-01 cells (5 × 10^5^ cells) into the left and right armpits of nude mice. As shown in Fig. 2A and B, the tumor formation rate in the adherent culture group was 100%, whereas it was only 50% in the suspension culture group. In addition, the CSC-like CTCs exhibited a longer latency period before tumor formation, slower tumor growth, and lower tumor weights in the nude mice (Fig. 2C–E). Figure 2F indicates that there were no significant differences in the morphological characteristics of the tumor tissues between the two groups, with both displaying high cell density. However, the proportion of Ki-67-positive cells, which are indicative of proliferative activity, was notably lower in the CSC-like CTC group (Fig. 2G). These results collectively indicate that CSC-like CTCs possess reduced tumorigenicity and tumor growth potential in nude mice.

### CSC-like CTCs are insensitive to chemotherapy drugs in vitro

**Fig. 3:**
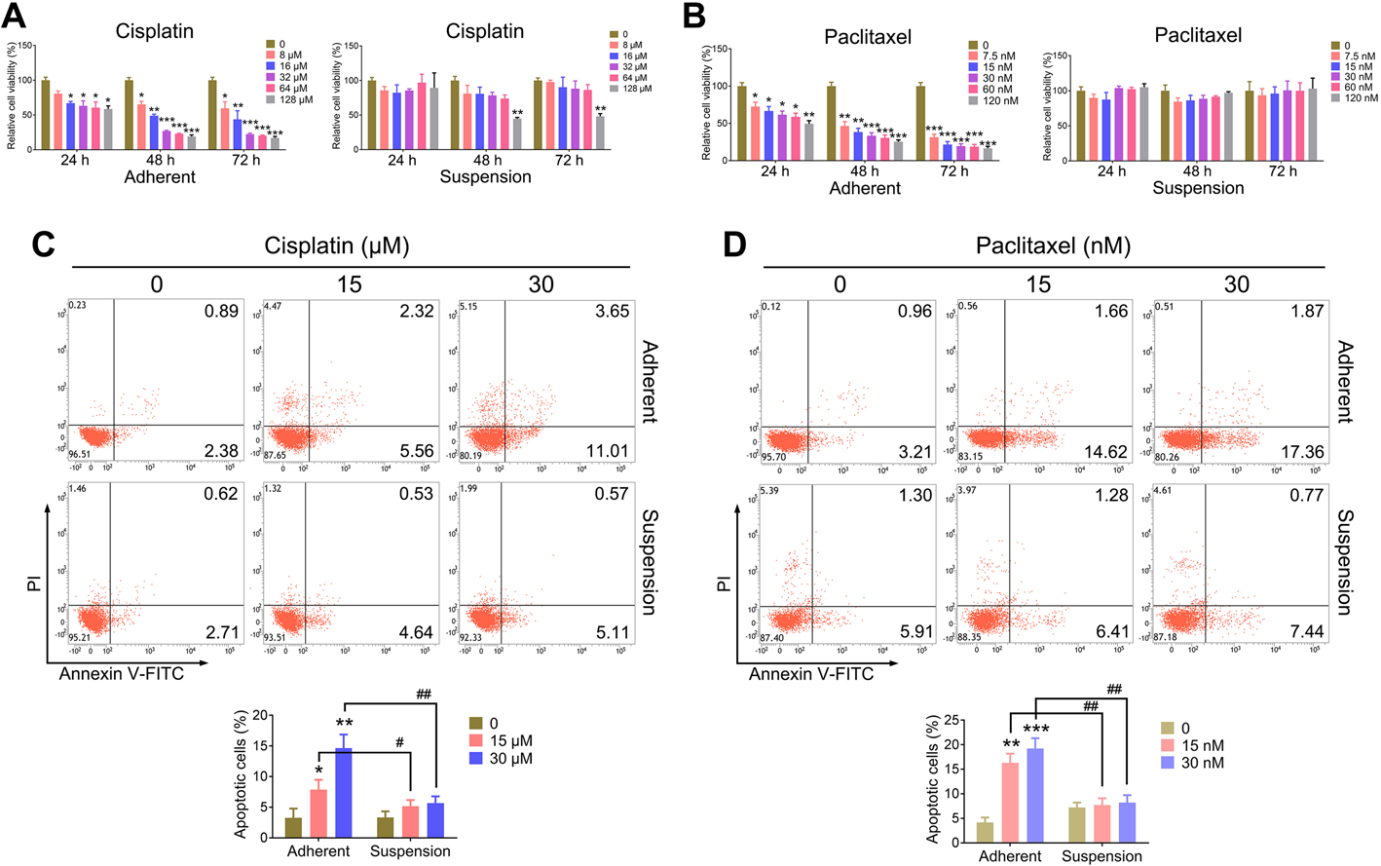
Comparison of the sensitivity of CTC-TJH-01 cells to chemotherapeutic drugs in adherent and suspension cultures in vitro. **A** Comparison of the drug sensitivity of adherent and suspension-cultured CTC-TJH-01 cells to cisplatin. **B** Comparison of the drug sensitivity of adherent and suspension cultured CTC-TJH-01 cells to paclitaxel. **C** Adherent and suspension-cultured CTC-TJH-01 cells were treated with cisplatin (0, 15, and 30 μM) for 48 h. Flow cytometry was performed to determine the degree of CTC-TJH-01 cell apoptosis. **D** Adherent and suspension-cultured CTC-TJH-01 cells were treated with paclitaxel (0, 15, and 30 nM) for 48 h. Flow cytometry was performed to determine the degree of CTC-TJH-01 cell apoptosis. Each bar represents the mean ± SD of three separate experiments. *P < 0.05; **P < 0.01; ***P < 0.001

The presence of CSCs contributes significantly to clinical chemoresistance [27]. To evaluate the chemosensitivity of CSC-like CTCs to cisplatin and paclitaxel, we performed experiments with adherent and suspension-cultured CTC-TJH-01 cells. We utilized a CCK-8 assay and an annexin V-FITC/PI apoptosis assay to assess cell proliferation and apoptosis. Our findings revealed that both cisplatin and paclitaxel effectively inhibited the proliferation of adherent CTC-TJH-01 cells in a time- and dose-dependent manner (Fig. 3A, B), with IC_50_ values of 14.95 ± 3.28 μM for cisplatin and 5.37 ± 1.06 nM for paclitaxel at 72 h. In contrast, CTC-TJH-01 cells in suspension exhibited strong resistance to these drugs. At high concentrations, cisplatin was cytotoxic to cell clusters only at concentrations up to 128 μM, whereas paclitaxel failed to exhibit significant cytotoxic effects even at 120 nM (Fig. 3A, B). In addition, the results of the apoptosis assays indicated that while cisplatin and paclitaxel significantly induced apoptosis in adherent CTC-TJH-01 cells, they had minimal effects on cells in suspension culture (Fig. 3C, D). These findings demonstrate that CSC-like CTCs are highly resistant to chemotherapy with cisplatin and paclitaxel.

### CSC-like CTCs are resistant to cisplatin in vivo

**Fig. 4:**
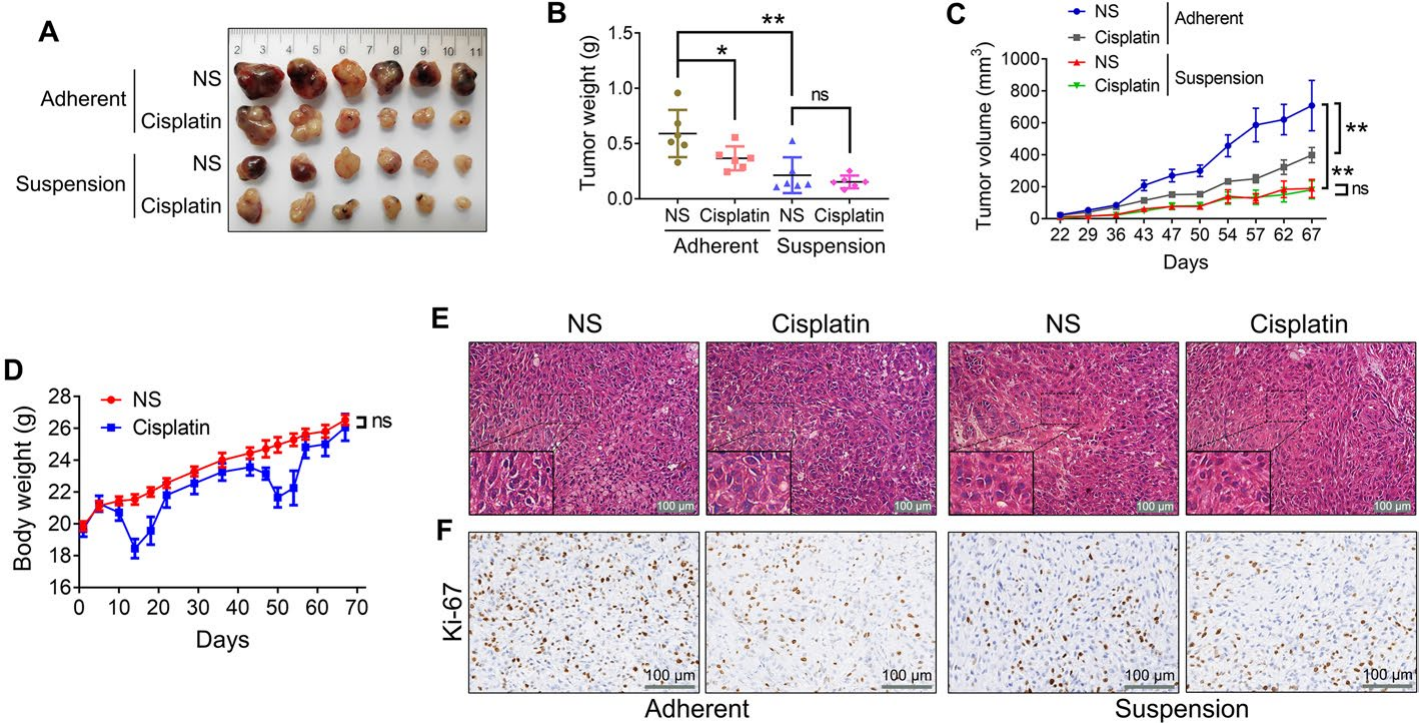
Comparison of the sensitivity of CTC-TJH-01 cells to cisplatin in adherent and suspension cultures in vivo. **A** Adherent and suspension-cultured CTC-TJH-01 cells (1 × 10^6^ cells) were subcutaneously transplanted into nude mice, which were subsequently treated with cisplatin (2 mg/kg) for 5 continuous days, for a total of two treatments. The tumors were excised and photographed on the 67th day (*n* = 6). **B** Tumor weight was measured with an electronic balance. **C** Tumor growth was measured with a digital caliper and tumor growth curves were drawn. **D** Mouse body weights were measured twice a week. **E** HE staining of CTC-TJH-01 cell xenografts. Scale bar, 100 μm. **F** Immunohistochemical analyses of Ki-67 expression levels in CTC-TJH-01 cell xenograft tumor tissues. Scale bar, 100 μm. Each bar represents the mean ± SEM of three separate experiments. *P < 0.05; **P < 0.01; ***P < 0.001

To further assess the drug sensitivity of CSC-like CTCs in vivo, we subcutaneously inoculated the same number of CTC-TJH-01 cells (1 × 10^6^ cells) after adherent and suspension culture into the left and right armpits of nude mice. Compared with those derived from adherent cultures, we observed that tumors derived from CTC-TJH-01 cells in suspension culture were smaller, lighter, and grew more slowly (Fig. 4A–C). Notably, while cisplatin (2 mg/kg) significantly inhibited the growth of xenografts from adherent-cultured CTC-TJH-01 cells, as evidenced by reduced tumor volume and weight, it did not significantly affect tumors from cells in suspension culture (Fig. 4A–C). Furthermore, cisplatin treatment notably decreased the body weight of the mice, although their weight quickly recovered after treatment ended (Fig. 4D). Histological examination through HE and immunohistochemical staining revealed an increase in vacuolization within the tumor cells and a significant reduction in Ki-67 protein expression, indicating decreased proliferation in tumors from the adherent culture group following cisplatin treatment (Fig. 4E, F). However, these changes were not observed in the suspension culture group. These findings collectively indicate that CSC-like CTCs exhibit significant resistance to cisplatin in vivo.

### The CDH17-YAP pathway is activated in CSC-like CTCs

**Fig. 5:**
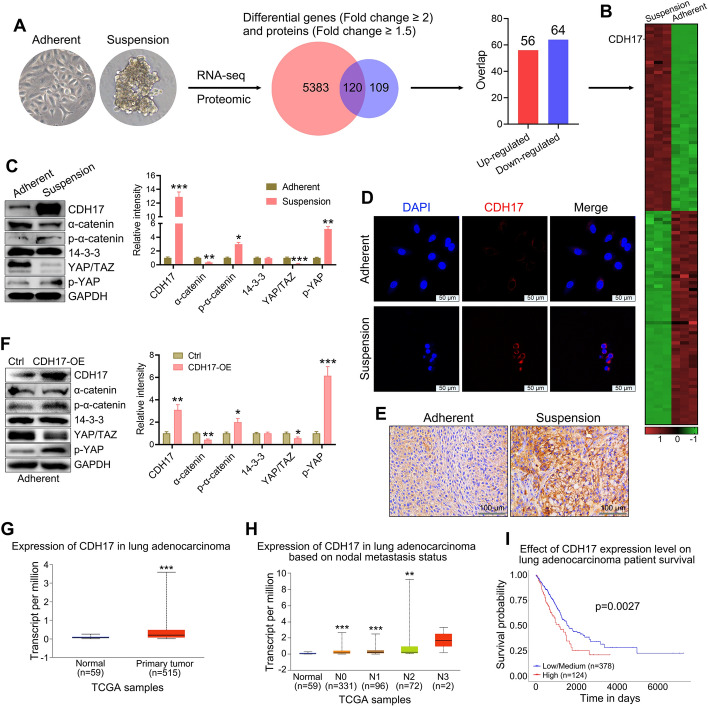
CDH17 is highly expressed in suspension-cultured CTC-TJH-01 cells. **A** Transcriptomic and proteomic analysis of differentially expressed genes and proteins between adherent and suspension-cultured CTC-TJH-01 cells. **B** Heatmap of differentially expressed genes. **C** Western blot analysis of CDH17 and YAP signaling pathway protein expression in adherent and suspension-cultured CTC-TJH-01 cells. **D** Immunofluorescence analysis of CDH17 protein expression in adherent and suspension-cultured CTC-TJH-01 cells. Scale bar, 50 μm. **E** Immunohistochemical analysis of CDH17 protein expression in adherent and suspension-cultured CTC-TJH-01 cell xenograft tumors. Scale bar, 100 μm. **F** Overexpression of the CDH17 gene in adherent-cultured CTC-TJH-01 cells. The left panel displays the western blot analysis, whereas the right panel presents the quantification of protein expression levels. **G** Expression of the CDH17 gene in lung adenocarcinoma and adjacent tissues. **H** Expression of the CDH17 gene in lung adenocarcinoma tissues at different clinical stages. **I** Survival analysis of patients with lung adenocarcinoma stratified by the expression level of the CDH17 gene. Each bar represents the mean ± SD of three separate experiments. *P < 0.05; **P < 0.01; ***P < 0.001

To further elucidate the molecular mechanisms underlying the cancer stem-like characteristics of CTCs following suspension culture, we analyzed the differentially expressed genes and proteins in CTC-TJH-01 cells cultured under both adherent and suspension conditions. As shown in Fig. 5A, there were 120 overlapping differentially expressed genes (DEGs) and proteins, 56 of which were upregulated and 64 of which were downregulated (suspension versus adherent). Notably, the expression of CDH17 was significantly elevated (Fig. 5B). Further examination of CDH17 in both cells and tumor tissues revealed a marked increase in the percentage of CTC-TJH-01 cells cultured in suspension (Fig. 5C–E). In addition, while the protein expression of α-catenin and YAP/TAZ decreased, the protein expression of phospho-α-catenin and phospho-YAP significantly increased in suspended CTC-TJH-01 cells (Fig. 5C). Furthermore, overexpression of the CDH17 gene in adherent-cultured CTC-TJH-01 cells resulted in significant upregulation of phospho-α-catenin and phospho-YAP protein expression. (Fig. 5F). Analysis of the TCGA database revealed that, compared with that in normal lung tissue, the expression level of CDH17 in lung cancer tissue was substantially higher and was positively correlated with the clinical stage of lung cancer (Fig. 5G, H). Importantly, patients with lung cancer with elevated CDH17 expression in tumor tissues had poorer survival outcomes (Fig. 5I). These results suggest that the enhanced cancer stemness observed in suspended CTCs may be attributed to activation of the CDH17-YAP signaling pathway.

### CDH17 knockdown decreased cancer stemness, promoted tumor growth, and increased the sensitivity of tumor cells to cisplatin

**Fig. 6:**
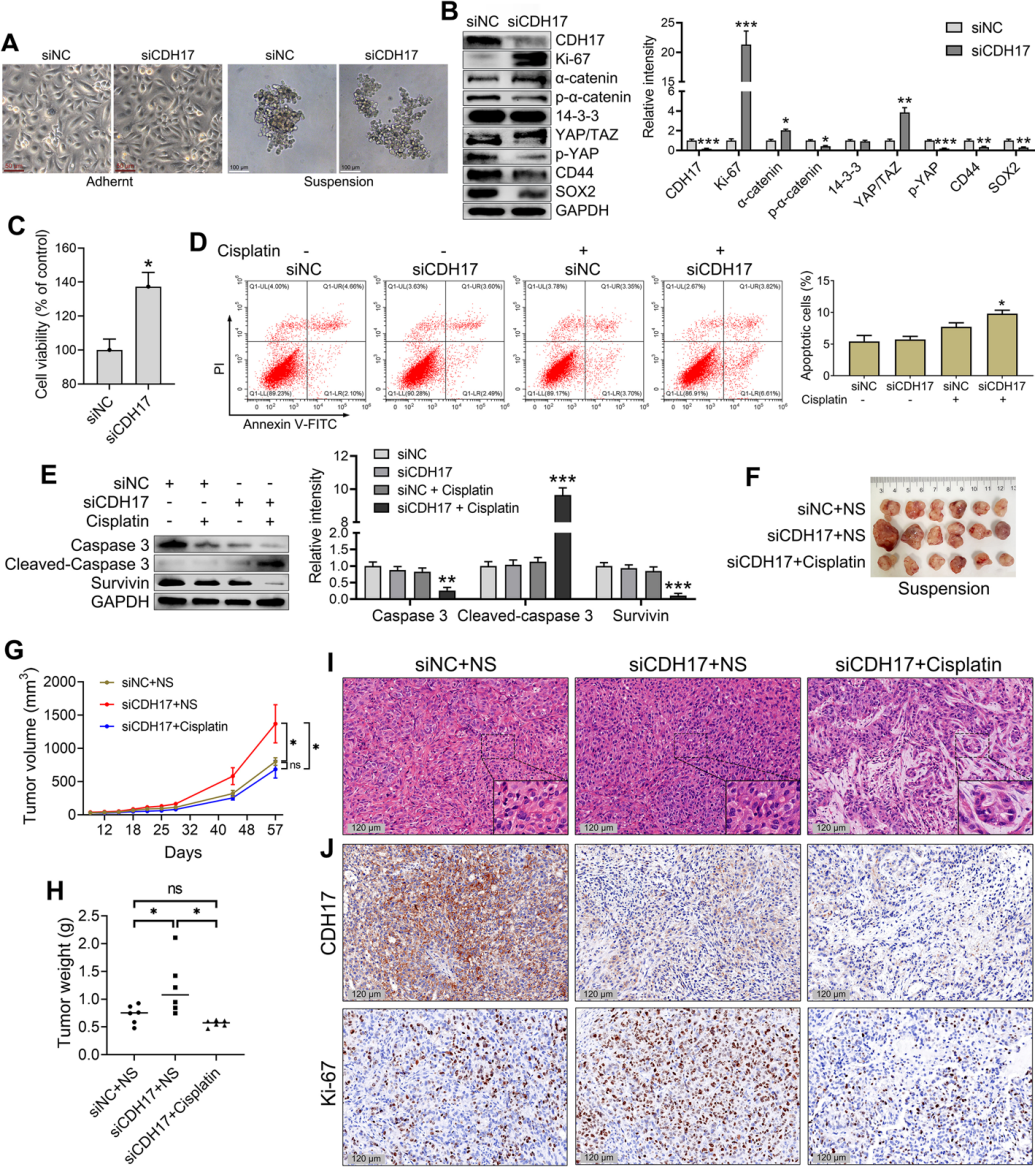
Knockdown of CDH17 promotes the proliferation and cisplatin sensitivity of CTC-TJH-01 cell clusters. **A** Morphological observation of CTC-TJH-01 cells after knockdown of the CDH17 gene. **B** Detection of YAP signaling pathway related proteins after CDH17 knockdown via western blotting. **C** The proliferative activity of CTC-TJH-01 cells after CDH17 knockdown was detected via a CCK-8 assay. **D** When the CDH17 gene was knocked down in suspension-cultured CTC-TJH-01 cells treated with cisplatin, apoptosis was detected by flow cytometry. **E** When the CDH17 gene was knocked down in suspension-cultured CTC-TJH-01 cells treated with cisplatin, the expression of caspase 3 and survivin was analyzed by western blotting. **F** A representative image of a tumor excised 57 days after subcutaneous transplantation of a suspension of cultured CTC-TJH-01 cells (1 × 10^6^ cells) into NOD-SCID mice (*n* = 6). **G** Tumor volume was measured with a digital caliper. **H** Tumor weight was measured with an electronic balance. **I** HE staining of CTC-TJH-01 cell cluster xenograft tumor tissues. Scale bar, 120 μm. **J** Immunohistochemical analyses of CDH17 and Ki-67 expression levels in CTC-TJH-01 cell cluster xenograft tumor tissues. Scale bar, 120 μm. Each bar represents the mean ± SD of three separate experiments. *P < 0.05; **P < 0.01; ***P < 0.001

To further explore the role of the CDH17-YAP pathway in the cancer stemness and drug resistance of suspended CTCs, we utilized siRNA to downregulate CDH17 protein expression in CTC-TJH-01 cells. As shown in Fig. 6A, after knockdown of the CDH17 gene, there was no obvious effect on the morphology of adherent CTC-TJH-01 cells, but it markedly reduced the tightness of CTC-TJH-01 cell clusters in the suspended state. In addition, this treatment led to significant reductions in phospho-α-catenin, phospho-YAP, CD44, and SOX2 levels, whereas the expression levels of Ki-67, α-catenin, and YAP/TAZ were notably increased (Fig. 6B). Importantly, CDH17 knockdown significantly enhanced both the proliferation activity and cisplatin sensitivity of the suspended CTCs (Fig. 6C–E). Furthermore, CDH17 knockdown facilitated cisplatin-induced apoptosis in CTC-TJH-01 cell clusters, as indicated by a significant decrease in survivin protein levels and an increase in cleaved caspase-3 protein expression (Fig. 6D, E).

We also evaluated the impact of CDH17 knockdown on the growth of CSC-like CTC xenograft tumors and their sensitivity to cisplatin in vivo. CDH17 knockdown markedly increased the growth of tumor xenografts and significantly enhanced the inhibitory effect of cisplatin on tumor growth (Fig. 6F–H). However, there was no significant difference in tumor volume or weight between the CDH17 knockdown plus cisplatin treatment group and the control group (Fig. 6G, H). Figure 6J shows that the percentage of Ki-67 positive cells in the tumor tissues significantly increased following CDH17 knockdown. Additionally, cisplatin treatment substantially increased the number of irregular cell voids in tumor tissue and significantly reduced the proportion of Ki-67-positive cells.

These findings underscore that CDH17 is a key regulator of tumor stemness and chemoresistance in CTCs. As summarized in Table 1, tumor growth kinetics are closely linked to chemotherapy resistance; tumors with a low growth rate are more resistant to chemotherapy, whereas those with a high growth rate only experience a reduction in growth rate following chemotherapy treatment. These findings suggest that identifying metastases with a low proliferation rate may be crucial to understanding drug resistance and the cause of patient mortality.

**Table 1:** Tumor growth kinetics and chemoresistance in different cellular states

Culture method	Group	Slope	Equation
Adherent	NS	16.7	*Y* = 16.7 × *X* − 447.0
	Cisplatin	8.2	*Y* = 8.2 × *X* − 209.2
Suspension	NS	4.4	*Y* = 4.4 × *X* − 114.8
	Cisplatin	4.1	*Y* = 4.1 × *X* − 109.6
Suspension	siNC + NS	14.5	*Y* = 14.5 × *X* − 192.5
	siCDH17 + NS	25.5	*Y* = 25.5 × *X* − 367.2
	siCDH17 + Cisplatin	12.5	*Y* = 12.5 × *X* − 178.9

*NS* normal saline, *NC* negative control (scrambled siRNA)

## Discussion

Our research revealed that CTCs in suspension spontaneously form spheres and exhibit CSC-like properties, diminished proliferative activity, and increased chemoresistance. Notably, CTCs with low proliferation rates demonstrate reduced tumorigenicity and slower tumor growth, resulting in decreased chemotherapy sensitivity in mice. The inactivation of the CDH17-YAP pathway in CTC clusters significantly increases both the proliferation and chemotherapy sensitivity of these clusters. Chemotherapy primarily moderates tumor growth rates. These insights advance our understanding of the chemoresistance traits of CTCs and their implications for tumor biology.

CTCs are shed from primary or metastatic tumors and serve as the “seeds” of tumor metastasis [8]. These cells exhibit significant phenotypic plasticity, allowing for the detection of CTCs at various stages of EMT in peripheral blood [14]. Previous research has shown that CTCs derived from peripheral blood exhibit dormancy, cancer stemness, robust drug resistance, tumorigenicity, and the ability to metastasize to the lung [23]. Moreover, evidence suggests that CTC clusters have significantly greater metastatic potential than single CTCs [11]. In this study, we utilized our established human circulating lung tumor cell line, CTC-TJH-01, to further explore the unique biological properties of these clusters. CTC-TJH-01 cells are derived from the peripheral blood of a patient with lung adenocarcinoma. Consistent with the findings of Yu et al. [28], our CTC-TJH-01 cells in suspension formed clusters, exhibited high levels of CD44 and SOX2, and showed reduced proliferation compared with that of adherent cells. This finding aligns with that of Gkountela et al., who reported hypomethylation at stemness- and proliferation-associated transcription factor-binding sites in breast cancer CTC clusters [29]. Notably, compared with primary lung cancer A549 and 95-D cells, CTC-TJH-01 cells cultured under adherent conditions also displayed drug resistance, stem cell-like traits, and immune evasion properties, as previously reported [23]. The tumorigenic capacity of CTC-TJH-01 cell clusters in nude mice was significantly diminished; these cells took longer to form tumors, and the tumor growth rate decreased. This observation echoes findings from Park et al., who noted that while the proliferation of MDA-MB-468 breast cancer cells in suspension culture was slower, their tumorigenic and metastatic abilities were notably enhanced in vivo [30]. We hypothesize that the enhanced cancer stemness of CTC-TJH-01 cell clusters may coincide with a dormant state, leading to their diminished tumorigenic and tumor growth capabilities. These findings suggest that various growth environments critically impact the proliferation, cancer stemness, and tumorigenic potential of CTCs.

Cancer cells with a stem cell-like phenotype are primarily responsible for tumor recurrence, chemoresistance, and metastasis postchemotherapy, significantly impacting the survival of patients with cancer [31]. Chemotherapy tends to enrich CSC populations in tumors, and inhibiting key CSC markers, such as CD44, SOX2, and ALDH1, can enhance chemosensitivity [32]. The overexpression of ABC transporters, cell dormancy, and enhanced DNA damage repair mechanisms are pivotal in mediating the resistance of CSCs to chemotherapy [31]. A recent study by França et al., published in Nature, explored how cancer cells adapt to treatments through a mechanism known as the resistance continuum. This process involves crucial transitions between different cell states, allowing cancer cells to adjust to and survive drug treatments. This study highlights the potential of targeting these cell-state transitions as a novel strategy for cancer therapy [33]. Our findings indicate that stem cell-like CTC-TJH-01 cell clusters exhibit reduced proliferative activity and are resistant to the chemotherapeutic agents, cisplatin and paclitaxel. In addition, xenografts derived from these stem cell-like CTC-TJH-01 cell clusters also displayed resistance to cisplatin in vivo. However, cisplatin significantly inhibited the growth of conventional CTC-TJH-01 cell xenografts, which exhibited increased growth. Furthermore, our previous studies have shown that CTC-TJH-01 cells exhibit lower proliferative activity than A549 and 95-D cells, yet they are more resistant to cisplatin and taxotere [23]. These observations suggest that the proliferation rate of tumor cells is a critical determinant of their sensitivity to chemotherapy drugs.

Recently, a developmental constraint model has been proposed that posits that cancer cell states and tumor heterogeneity are defined by the expression of gene modules. This model suggests that cancer cells are highly plastic and capable of transitioning between various states—such as stem cell-like, migratory, and proliferative states—with chromatin remodeling playing a crucial role in these transitions [34]. We sought to elucidate the mechanisms underlying the cancer stemness and drug resistance of CTC-TJH-01 cell clusters through comprehensive “omics” studies. Our analyses revealed significant upregulation of the CDH17 gene and protein in these cell clusters. CDH17 is a transmembrane protein in the cadherin family, with extracellular, transmembrane, and intracellular domains. The extracellular domain mediates cell–cell adhesion, and the intracellular domain participates in intracellular signal transduction and the regulation of cell morphology [35]. Previous research has indicated that high CDH17 expression in tumor tissues is correlated with poorer prognosis and survival in patients with colorectal cancer, cholangiocarcinoma, and hepatocellular carcinoma [17, 18]. Utilizing the TCGA database, we also revealed that elevated CDH17 expression in lung cancer tissues was associated with advanced disease progression and reduced patient survival. CDH17 is known to activate the Wnt/β-catenin signaling pathway, promoting growth and metastasis in hepatocellular and colorectal cancers [17]. This insight has led researchers to explore CAR-T cells that target CDH17 for the treatment of colorectal, small cell lung, and gastric cancer [36, 37], suggesting that CDH17 may similarly mediate stemness and chemoresistance in CTC-TJH-01 cell clusters.

The Hippo signaling pathway, which is crucial for regulating organ size and maintaining stemness in various cancers [19], involves YAP, a key effector that partners with TAZ to control gene expression. Disruptions in YAP/TAZ phosphorylation are linked to the self-renewal, proliferative capacity, and increased chemoresistance of CSCs [38]. Our findings confirmed the activation of the Hippo pathway in CTC-TJH-01 cell clusters. We speculate that in the suspended CTC-TJH-01 cell clusters, the increase in the protein level of CDH17 and the reduction in the total protein levels of α-catenin and YAP/TAZ may be regulated mainly by epigenetics. In suspended CTC-TJH-01 cells, the CDH17 protein is overexpressed. The CDH17 proteins on the surfaces of adjacent cells interact to form cell–cell adhesion connections, which then activate intracellular α-catenin, β-catenin, and p120-catenin, and their phosphorylation [17]. The protein α-catenin can inhibit YAP by restricting its localization in a complex with 14-3-3. This complex also protects YAP from dephosphorylation by the PP2A phosphatase [39]. In addition, CDH17 may also regulate the expression of the CD44 protein through the WNT/β-catenin signaling pathway and then regulate YAP/TAZ signaling through the CD44 protein [17, 40]. Notably, CDH17 knockdown not only significantly inactivated this pathway but also diminished cancer stemness and enhanced the proliferation and chemosensitivity of these clusters. The overexpression of the CDH17 gene in adherent-cultured CTC-TJH-01 cells significantly activated this pathway. Furthermore, CDH17 knockdown markedly increased the growth of CTC-TJH-01 xenografts and their sensitivity to cisplatin. These observations underscore that the tumor cell growth rate is closely linked to chemotherapeutic sensitivity. Importantly, we observed that CTC-TJH-01 cells, when implanted in the lungs of mice, could develop metastatic lesions varying in size and proliferation rates [41]. These findings indicate that in the target organs of metastasis in patients with lung cancer, macro-metastatic lesions with relatively high growth rates are sensitive to treatment with chemotherapeutic drugs whereas micro-metastatic lesions with relatively slow growth rates coexist and are resistant to chemotherapy drugs. The sensitivity of macro-metastatic lesions to chemotherapy drugs derives from the relatively high proliferative activity of the cells at the periphery of the tumor, while the tumor cells in the middle layer are relatively quiescent and resistant to chemotherapy [42]. This is one of the important reasons why chemotherapy drugs can only reduce the growth rate of tumors. The continuous growth of these metastatic lesions with low growth rates and drug resistance eventually leads to disease progression and death.

## Conclusions

Our study provides pivotal insights into the biology of CTC clusters, clearly linking CSC-like traits with chemosensitivity. We established that the CDH17-YAP pathway is active in CTC-TJH-01 clusters and that its suppression can reduce their chemoresistance, although it does not halt tumor growth. Our findings underscore that chemotherapy predominantly lowers tumor growth rates, suggesting that reduced growth at metastatic sites may contribute to metastasis and increased mortality in patients with lung cancer.

## Supplementary Information

Additional file 1.

## Data Availability

All the data generated or analyzed during this study are included in this published article.

## Abbreviations

CTC: Circulating tumor cell
DTC: Disseminated tumor cell
CSC: Cancer stem cell
DFS: Disease-free survival
OS: Overall survival
PD-L1: Programmed death-ligand 1
CDH17: Cadherin-17
TCGA: The Cancer Genome Atlas
HE: Hematoxylin and eosin
EMT: Epithelial–mesenchymal transition
MET: Mesenchymal–epithelial transition
