# Supplementary material for: Regulating chemoresistance and cancer stemness: the CDH17-YAP pathway in distinct cellular states of lung cancer CTC clusters
Source: Cell Mol Biol Lett. 2025 Feb 24;30:23. doi: 10.1186/s11658-025-00696-9 (PMC11849222; doi:10.1186/s11658-025-00696-9)
Supplement: Supplementary file 1 — Additional file 1. [file 11658_2025_696_MOESM1_ESM.pdf]

Figure 1G-H

Ki-67		
a: used for Figure 1G	b	c
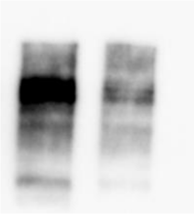<br>Adherent Suspension	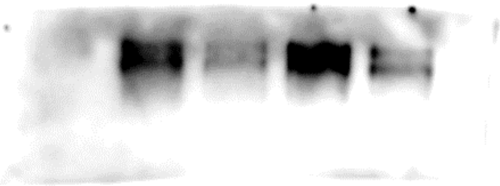<br>Adherent Suspension	Adherent Suspension

CDK1		
a: used for Figure 1G	b	c
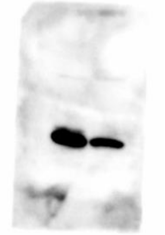<br>Adherent Suspension	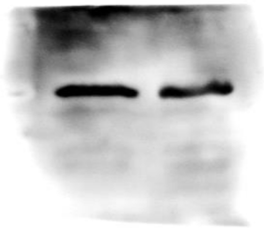<br>Adherent Suspension	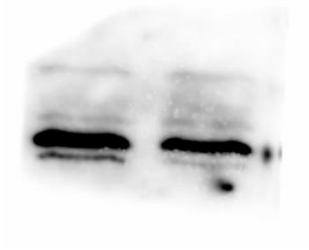<br>Adherent Suspension

CDK2		
a: used for Figure 1G	b	c
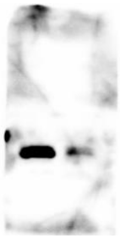<br>Adherent Suspension	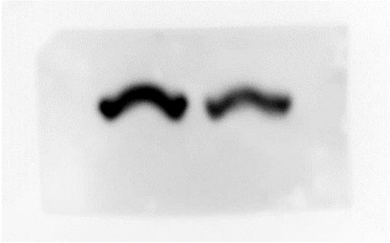<br>Adherent Suspension	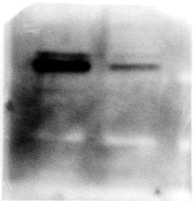<br>Adherent Suspension

CDK4		
a: used for Figure 1G	b	c
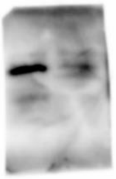<br>Adherent Suspension	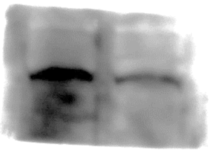<br>Adherent Suspension	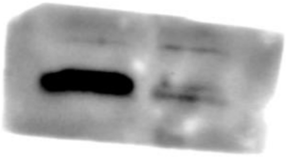<br>Adherent Suspension

CDK6		
a: used for Figure 1G	b	c
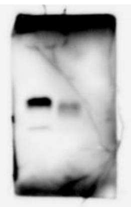 Adherent Suspension	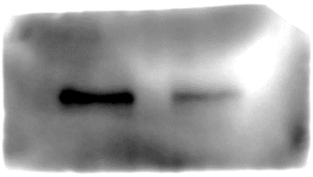 Adherent Suspension	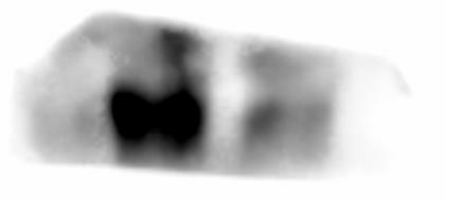 Adherent Suspension

Cyclin A2		
a: used for Figure 1G	b	c
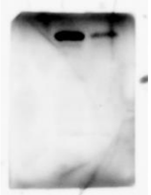 Adherent Suspension	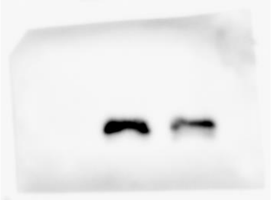 Adherent Suspension	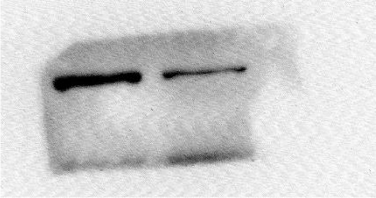 Adherent Suspension

Cyclin B1		
a: used for Figure 1G	b	c
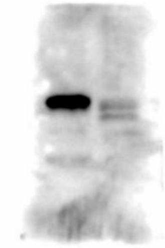 Adherent Suspension	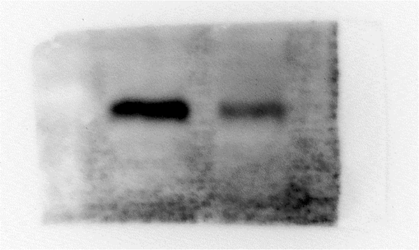 Adherent Suspension	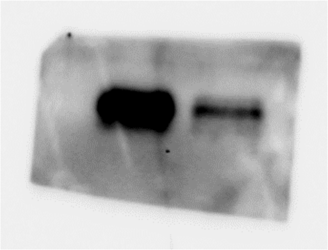 Adherent Suspension

Cyclin D1		
a: used for Figure 1G	b	c
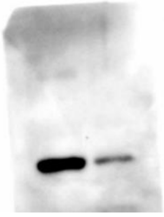 Adherent Suspension	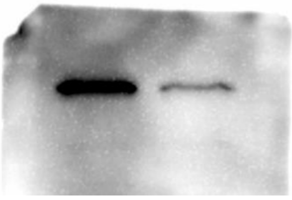 Adherent Suspension	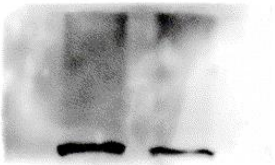 Adherent Suspension

Cyclin E1		
a: used for Figure 1G	b	c
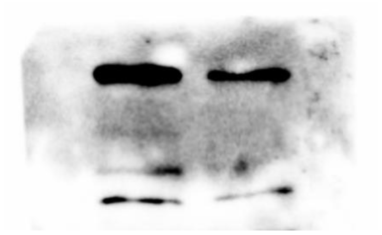 Adherent Suspension	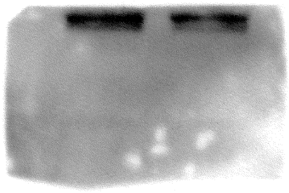 Adherent Suspension	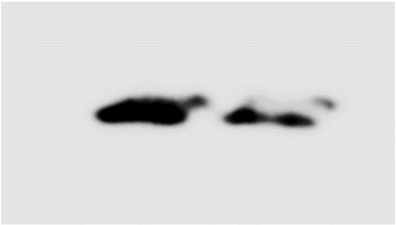 Adherent Suspension

ABCG2		
a: used for Figure 1H	b	c
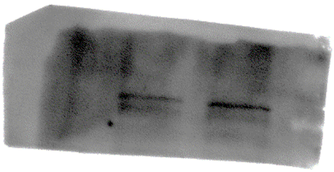 Adherent Suspension	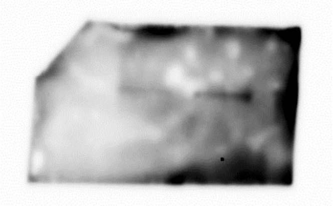 Adherent Suspension	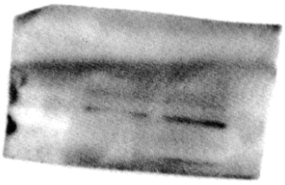 Adherent Suspension

ALDH1		
a: used for Figure 1H	b	c
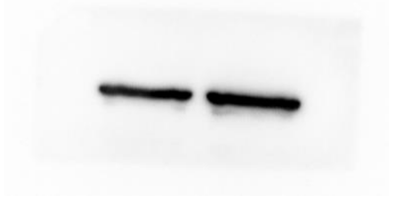 Adherent Suspension	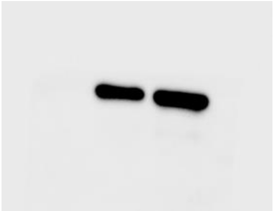 Adherent Suspension	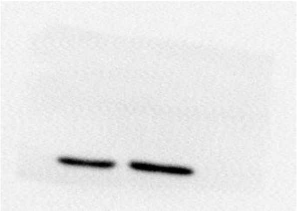 Adherent Suspension

CD44		
a: used for Figure 1H	b	c
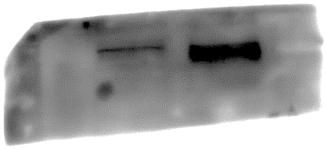 Adherent Suspension	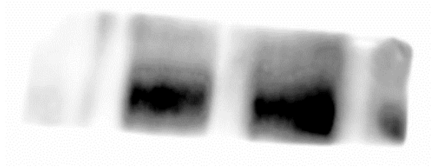 Adherent Suspension	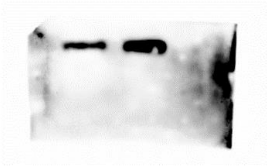 Adherent Suspension

CD133		
a: used for Figure 1H	b	c
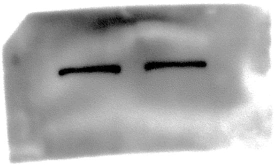<br>Adherent Suspension	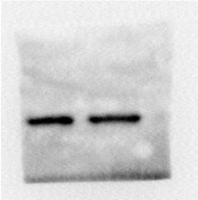<br>Adherent Suspension	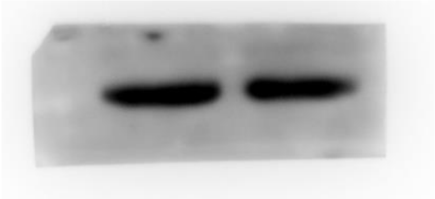<br>Adherent Suspension

OTC-4		
a: used for Figure 1H	b	c
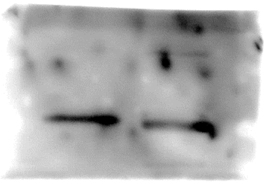<br>Adherent Suspension	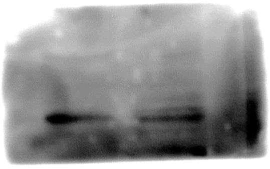<br>Adherent Suspension	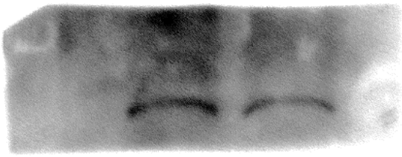<br>Adherent Suspension

SOX-2		
a: used for Figure 1H	b	c
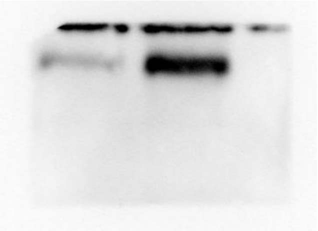<br>Adherent Suspension	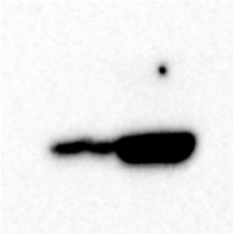<br>Adherent Suspension	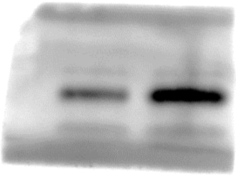<br>Adherent Suspension

GAPDH			
a: used for Figure 1G	b: used for Figure 1H	c	d
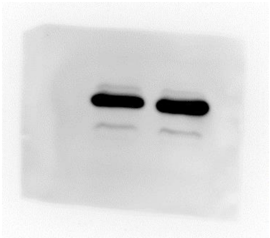<br>Adherent Suspension	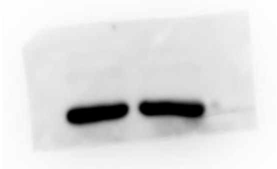<br>Adherent Suspension	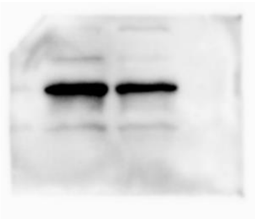<br>Adherent Suspension	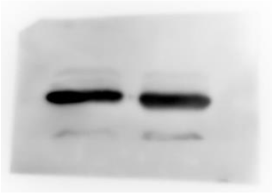<br>Adherent Suspension

Figure 5C

CDH17		
a: used for Figure 5C	b	c
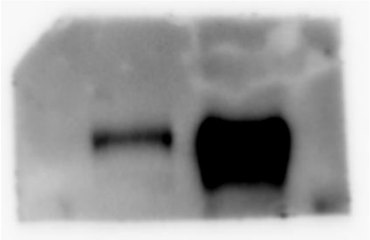 Adherent Suspension	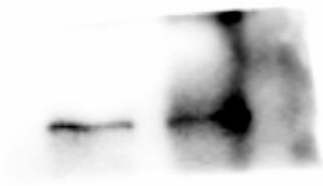 Adherent Suspension	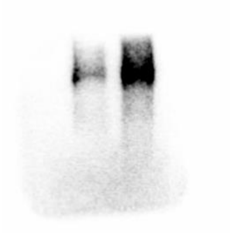 Adherent Suspension

α-catenin		
a: used for Figure 5C	b	c
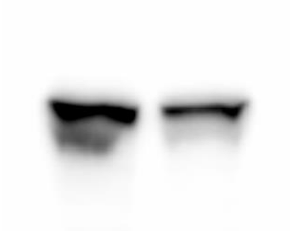 Adherent Suspension	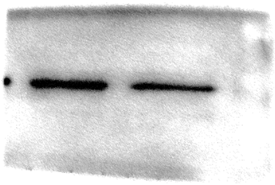 Adherent Suspension	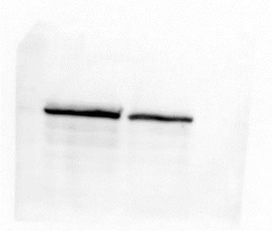 Adherent Suspension

p-α-catenin		
a: used for Figure 5C	b	c
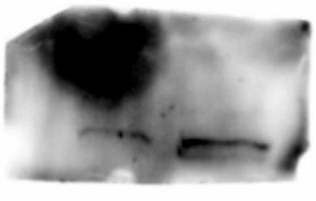 Adherent Suspension	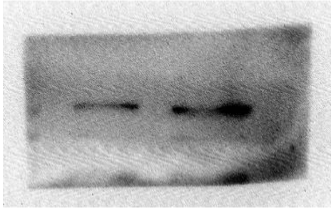 Adherent Suspension	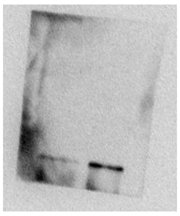 Adherent Suspension

14-3-3		
a: used for Figure 5C	b	c
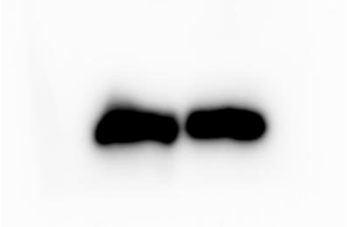 Adherent Suspension	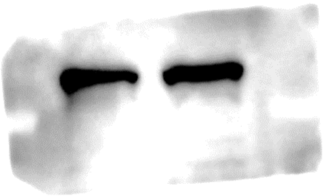 Adherent Suspension	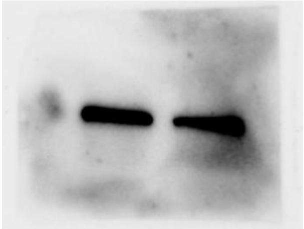 Adherent Suspension

YAP/TAZ		
a: used for Figure 5C	b	c
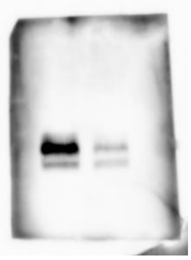<br>Adherent Suspension	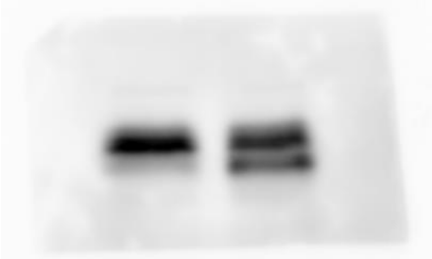<br>Adherent Suspension	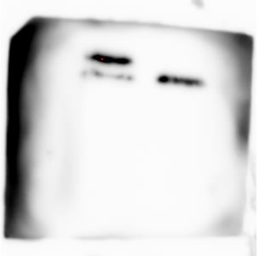<br>Adherent Suspension

p-YAP		
a: used for Figure 5C	b	c
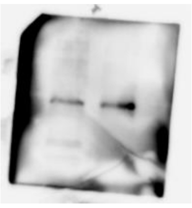<br>Adherent Suspension	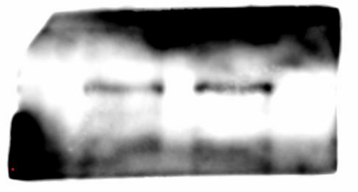<br>Adherent Suspension	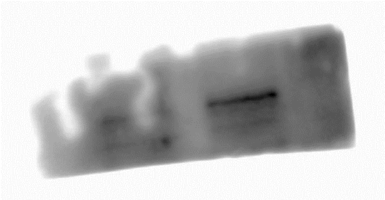<br>Adherent Suspension

GAPDH		
a: used for Figure 5C	b	c
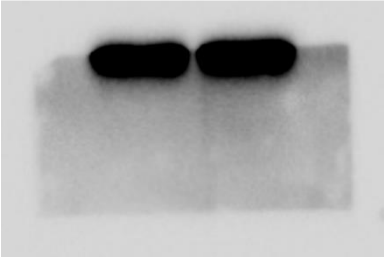<br>Adherent Suspension	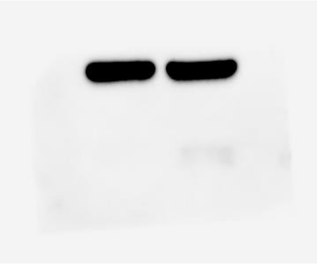<br>Adherent Suspension	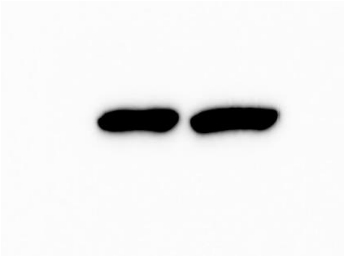<br>Adherent Suspension

Figure 5F

CDH17		
a: used for Figure 5F	b	c
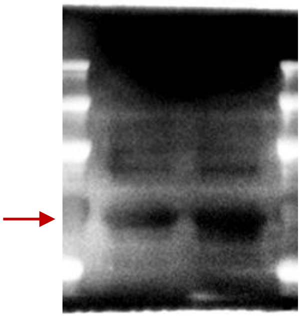<br>Ctrl CDH17-OE	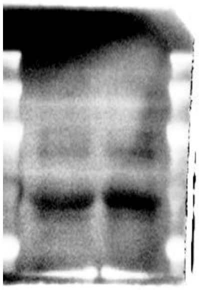<br>Ctrl CDH17-OE	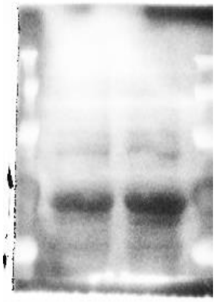<br>Ctrl CDH17-OE

α-catenin		
a: used for Figure 5F	b	c
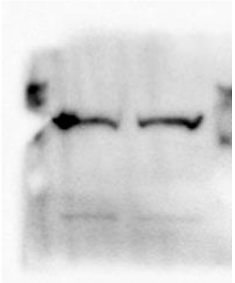<br>Ctrl CDH17-OE	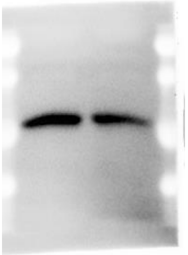<br>Ctrl CDH17-OE	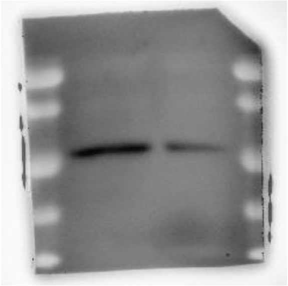<br>Ctrl CDH17-OE

p-α-catenin		
a: used for Figure 5F	b	c
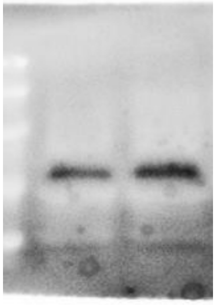<br>Ctrl CDH17-OE	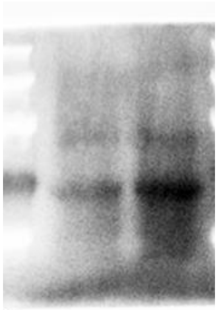<br>Ctrl CDH17-OE	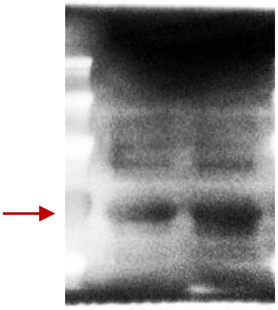<br>Ctrl CDH17-OE

14-3-3		
a: used for Figure 5F	b	c
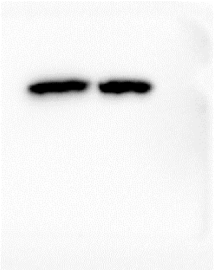<br>Ctrl CDH17-OE	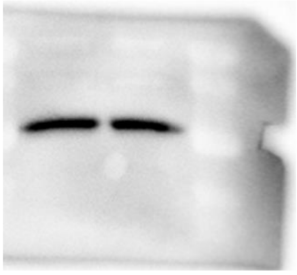<br>Ctrl CDH17-OE	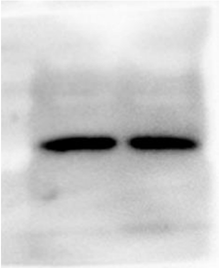<br>Ctrl CDH17-OE

YAP/TAZ		
a: used for Figure 5F	b	c
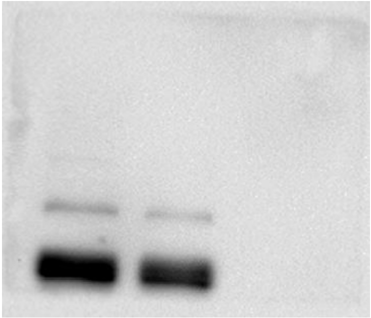<br>Ctrl CDH17-OE	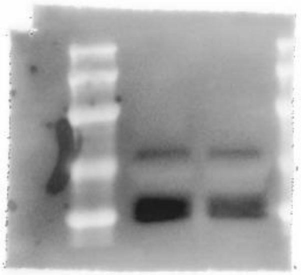<br>Ctrl CDH17-OE	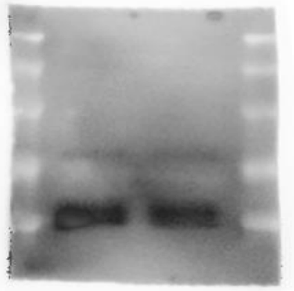<br>Ctrl CDH17-OE

p-YAP		
a: used for Figure 5F	b	c
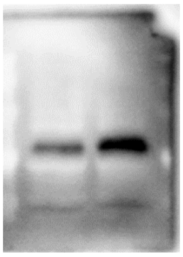<br>Ctrl CDH17-OE	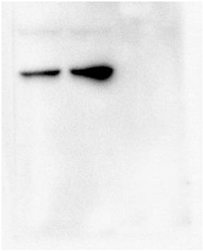<br>Ctrl CDH17-OE	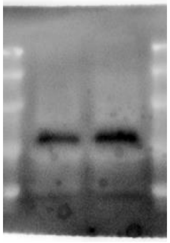<br>Ctrl CDH17-OE

GAPDH		
a: used for Figure 5F	b	c
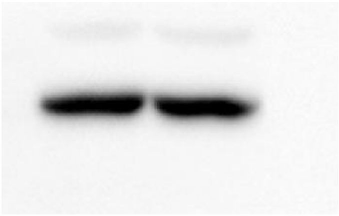<br>Ctrl CDH17-OE	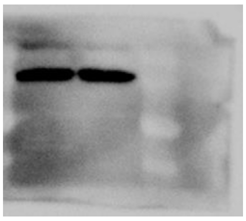<br>Ctrl CDH17-OE	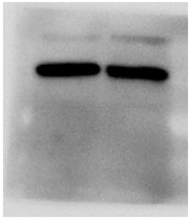<br>Ctrl CDH17-OE

Figure 6B

CDH17		
a: used for Figure 6B	b	c
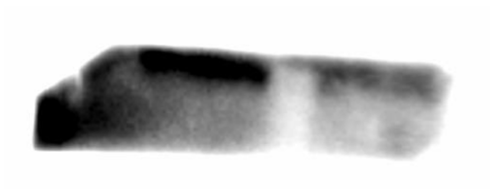 siNC siCDH17	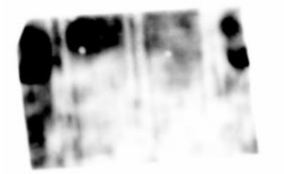 siNC siCDH17	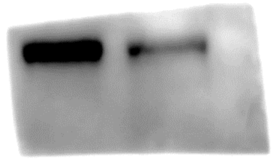 siNC siCDH17

Ki-67		
a: used for Figure 6B	b	c
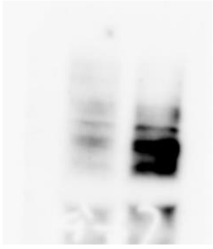 siNC siCDH17	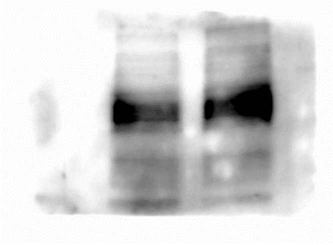 siNC siCDH17	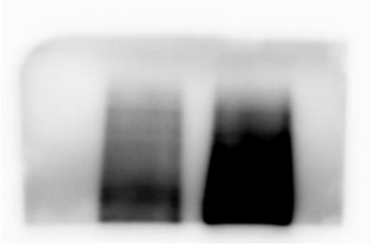 siNC siCDH17

α-catenin		
a: used for Figure 6B	b	c
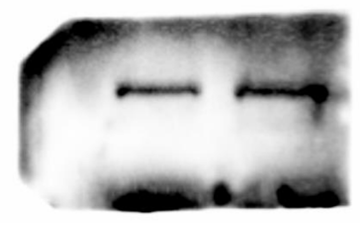 siNC siCDH17	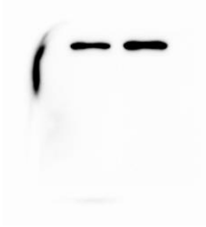 siNC siCDH17	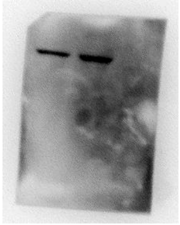 siNC siCDH17

p-α-catenin		
a: used for Figure 6B	b	c
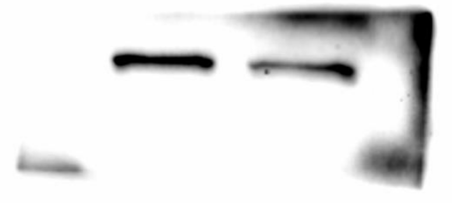 siNC siCDH17	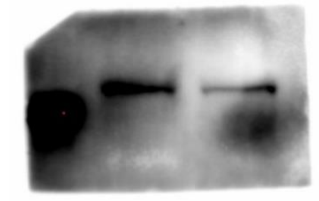 siNC siCDH17	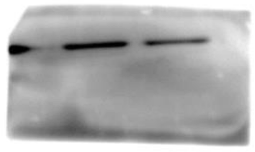 siNC siCDH17

14-3-3		
a: used for Figure 6B	b	c
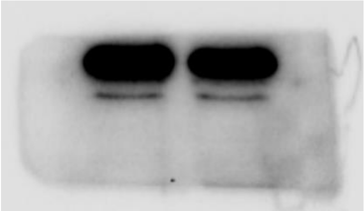<br>siNC siCDH17	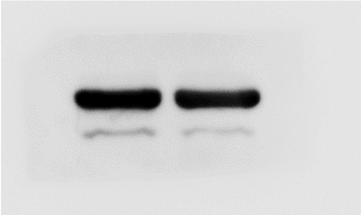<br>siNC siCDH17	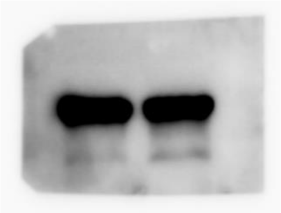<br>siNC siCDH17

YAP/TAZ		
a: used for Figure 6B	b	c
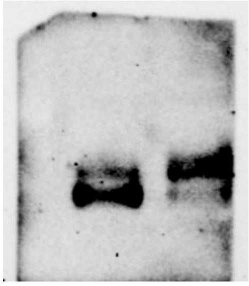<br>siNC siCDH17	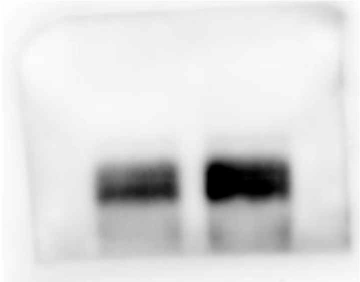<br>siNC siCDH17	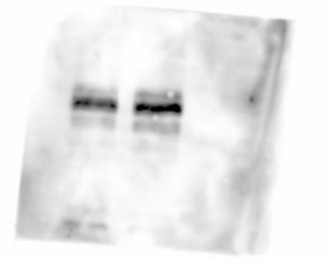<br>siNC siCDH17

p-YAP		
a: used for Figure 6B	b	c
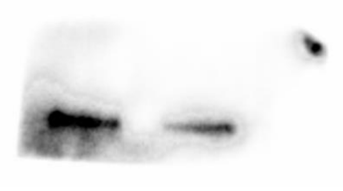<br>siNC siCDH17	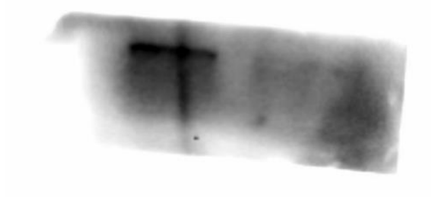<br>siNC siCDH17	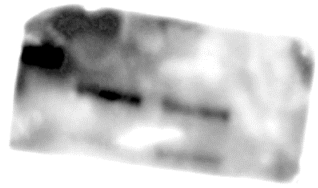<br>siNC siCDH17

CD44		
a: used for Figure 6B	b	c
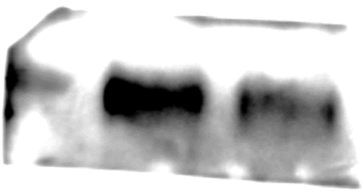<br>siNC siCDH17	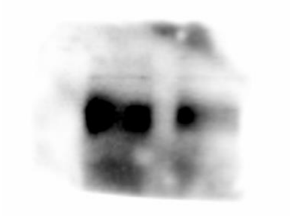<br>siNC siCDH17	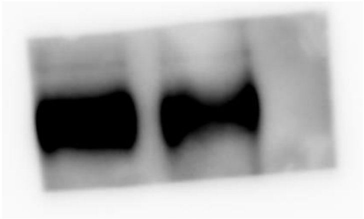<br>siNC siCDH17

<table>
<tr><td colspan="3">SOX2</td></tr>
<tr><td>a: used for Figure 6B</td><td>b</td><td>c</td></tr>
<tr><td colspan="2">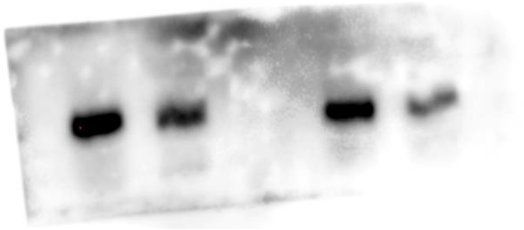
siNC siCDH17 siNC siCDH17</td><td>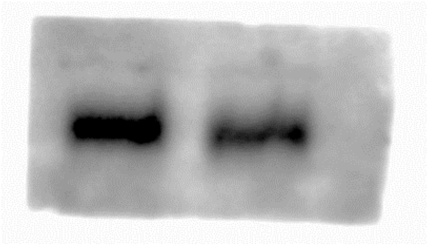
siNC siCDH17</td></tr>
</table>

<table>
<tr><td colspan="3">GAPDH</td></tr>
<tr><td>a: used for Figure 6B</td><td>b</td><td>c</td></tr>
<tr><td>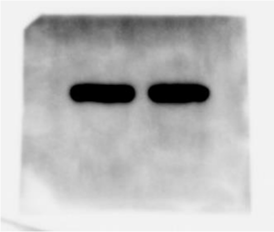
siNC siCDH17</td><td>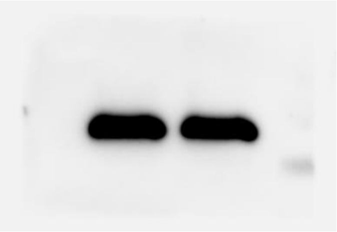
siNC siCDH17</td><td>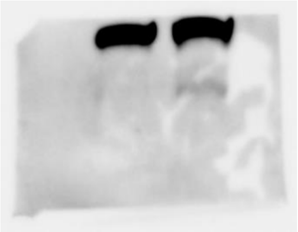
siNC siCDH17</td></tr>
</table>

Figure 6E

Caspase-3

a: used for Figure 6E	b	c
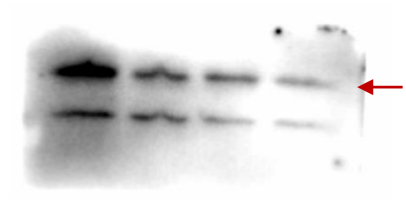	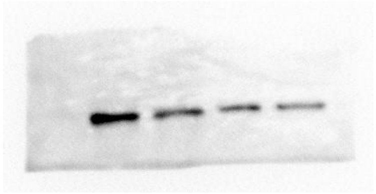	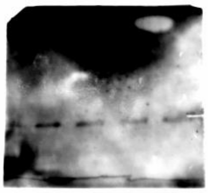
siNC + + - -<br>siCDH17 - - + +<br>Cisplatin - + - +	siNC + + - -<br>siCDH17 - - + +<br>Cisplatin - + - +	siNC + + - -<br>siCDH17 - - + +<br>Cisplatin - + - +

Cleaved caspase-3

a: used for Figure 6E	b	c
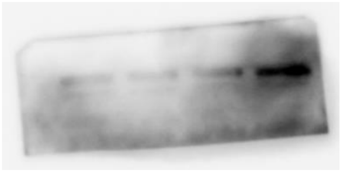	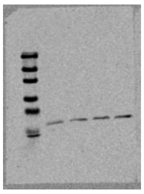	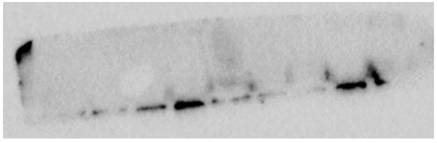
siNC + + - -<br>siCDH17 - - + +<br>Cisplatin - + - +	siNC + + - -<br>siCDH17 - - + +<br>Cisplatin - + - +	siNC + + - -<br>siCDH17 - - + +<br>Cisplatin - + - +

Survivin

a: used for Figure 6E	b	c
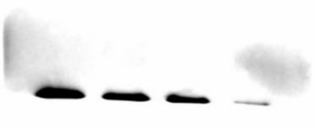	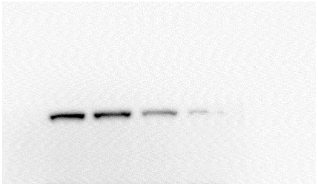	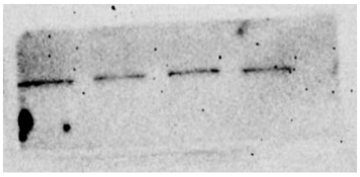
siNC + + - -<br>siCDH17 - - + +<br>Cisplatin - + - +	siNC + + - -<br>siCDH17 - - + +<br>Cisplatin - + - +	siNC + + - -<br>siCDH17 - - + +<br>Cisplatin - + - +

GAPDH

a: used for Figure 6E	b	c
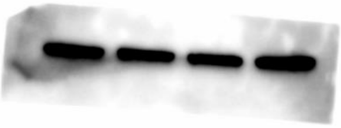	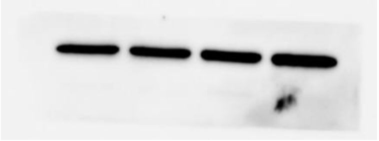	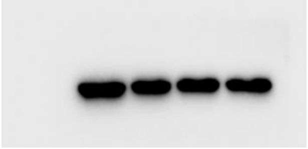
siNC + + - -<br>siCDH17 - - + +<br>Cisplatin - + - +	siNC + + - -<br>siCDH17 - - + +<br>Cisplatin - + - +	siNC + + - -<br>siCDH17 - - + +<br>Cisplatin - + - +
